# Thermal dependency of RAG1 self-association properties

**DOI:** 10.1186/1471-2091-9-5

**Published:** 2008-01-30

**Authors:** Pallabi De, Shuying Zhao, Lori M Gwyn, LeAnn J Godderz, Mandy M Peak, Karla K Rodgers

**Affiliations:** 1 Department of Biochemistry and Molecular Biology, The University of Oklahoma Health Sciences Center, Oklahoma City, Oklahoma 73190, USA; 2 Department of Molecular Genetics and Microbiology, University of Massachusetts Medical School, Worcester, Massachusetts 01655, USA; 3 Division of Biological Sciences, University of California, San Diego, La Jolla, California 92093, USA

## Abstract

**Background:**

Functional immunoglobulin and T cell receptor genes are produced in developing lymphocytes by V(D)J recombination. The initial site-specific DNA cleavage steps in this process are catalyzed by the V(D)J recombinase, consisting of RAG1 and RAG2, which is directed to appropriate DNA cleavage sites by recognition of the conserved recombination signal sequence (RSS). RAG1 contains both the active site and the RSS binding domains, although RAG2 is also required for DNA cleavage activity. An understanding of the physicochemical properties of the RAG proteins, their association, and their interaction with the RSS is not yet well developed.

**Results:**

Here, we further our investigations into the self-association properties of RAG1 by demonstrating that despite the presence of multiple RAG1 oligomers, only the dimeric form maintains the ability to interact with RAG2 and the RSS. However, facile aggregation of the dimeric form at physiological temperature may render this protein inactive in the absence of RAG2. Upon addition of RAG2 at 37°C, the preferentially stabilized V(D)J recombinase:RSS complex contains a single dimer of RAG1.

**Conclusion:**

Together these results confirm that the functional form of RAG1 in V(D)J recombination is in the dimeric state, and that its stability under physiological conditions likely requires complex formation with RAG2. Additionally, in future structural and functional studies of RAG1, it will be important to take into account the temperature-dependent self-association properties of RAG1 described in this study.

## Background

V(D)J recombination leads to the assembly of genes encoding for the immunoglobulin and T-cell receptors (TCR), and as a result is a fundamental process in the development of lymphocytes [1,2]. In the germline configuration, genes encoding the antigen-binding region of these receptors are divided into separate classes of genetic coding segments termed V (variable), D (diversity), and J (joining). Each gene segment is flanked by a recombination signal sequence (RSS) that contains both a conserved heptamer and a conserved nonamer sequence element separated by a spacer of either 12 ± 1 (12-RSS) or 23 ± 1 (23-RSS) base pairs. Recombination events are limited by the "12/23 rule" to those in which a pair of RSS participate, one 12-spacer signal and one 23-spacer signal [1,2].

The initial site-specific DNA cleavage steps in the V(D)J recombination mechanism are catalyzed by RAG1 and RAG2 [3]. Once the RAG proteins have created the double-strand breaks, the appropriate joining of both the coding and signal ends require factors that mediate nonhomologous DNA end joining [4]. There is evidence that the RAG proteins may prefer to assemble first on the 12-RSS to form the single RSS complex [5-8], followed by recruitment of the 23-RSS to form the paired complex [5,8,9]. The DNA-bending proteins, HMGB1 or HMGB2, facilitate formation of the latter complex [10]. The RAG proteins form DNA double strand breaks in two successive catalytic steps [3]. First, a DNA nick is generated between the coding gene segment and the heptamer of the RSS. Second, a DNA hairpin is formed by a transesterification reaction in which the 3'OH at the coding end is coupled to the phosphate group between the RSS and the gene segment on the opposing strand. The resulting products are a 5'-phosphorylated double-strand break at the signal end and a covalently closed hairpin at the coding end [3]. The macromolecular assemblies differ for each of the catalytic steps, with hairpin formation requiring the presence of both the 12 and 23-RSS in the paired complex, whereas nicking may be carried out on a 12-RSS in the single RSS complex [8]. Thus, to understand the parameters for both DNA cleavage steps, it is important to characterize both the single RSS and paired complexes. Significant questions remain about the architecture of both of these catalytically active complexes. For example, there have been conflicting reports concerning the stoichiometry of the RAG proteins in the V(D)J recombinase. Specifically, the stoichiometry of RAG1 was reported as dimer both in the single RSS complex [11,12] and in the paired complex [12]. Conversely, a separate study concluded that at least a trimer of RAG1 was present in both the single RSS and the paired complexes [13].

Both RAG proteins are required for catalytic activity. However, the core region of RAG1 (residues 384–1008; the minimal region required for V(D)J recombination activity) contains the active site motif and the relevant DNA binding domains that recognize the RSS heptamer and nonamer. RAG1 residues D600, D708, and E962 constitute the DDE motif active site, with these residues acting to coordinate the divalent metal cations essential for the DNA cleavage functions [14,15]. In addition, RAG1 residues 384–450 mediate DNA binding to the RSS nonamer [16,17], while the binding site for the RSS heptamer is located in the central domain (residues 528–760) [18]. The DNA binding capabilities of RAG1 alone may be significant since it is not clear if the V(D)J recombinase binds to the RSS as a pre-formed complex, or if RAG1 binds first, followed by RAG2. It is also not known if the total amount of RAG1 in developing lymphocytes is complexed with RAG2, or if there is a separate pool of free RAG1. In addition, RAG2 protein levels, but not RAG1, are cell cycle regulated, with increasing degradation occurring at the transition from G1 to the S phase [19]. Thus, there may be times in the cell cycle when only RAG1 is present. As a result it is important to determine the characteristics of RAG1 in the absence of RAG2.

RAG1, expressed and purified from bacteria, has been shown to exist in multiple, oligomeric forms [20,21]. However, little is known about RAG1 oligomerization interfaces since a high-resolution structure of core RAG1 has yet to be determined. Nevertheless, a recently reported crystal structure of a eukaryotic transposase, Hermes transposase, has yielded a feasible model for the overall topology of core RAG1 [22]. Similar to the RAG-mediated DNA cleavage mechanism, Hermes transposase catalyzes hairpin formation on the flanking DNA rather than the signal ends [23]. Significantly, the crystal structure of Hermes transposase revealed the presence of three structural domains [22], which may reflect how the three domains identified in core RAG1 [18] are oriented in the tertiary structure. Hermes was shown to be active as a dimer, but full activity required formation of a hexamer [22]. Likewise, characterization of the oligomeric properties of RAG1 is important to determine its requirement for full catalytic activity. To this end, previous studies demonstrated that core RAG1 was predominantly dimeric in solution [24], although higher order species, namely tetramer and octamer, were observed [20,21]. The relative proportions of tetrameric and octameric RAG1 increased significantly at ionic strengths approaching physiological conditions [21]. However, it was not clear if these higher order oligomeric forms of RAG1 directly contributed in the V(D)J recombination reaction.

In this study we investigated the self-association properties of RAG1 as a function of temperature, with an emphasis on the temperature range of 10–37°C. Dimer and octamer forms of RAG1, but not tetramer, were detectable over the entire temperature range studied. However, only the dimer species maintained interaction with the RSS and RAG2, and is thus the form likely present in the catalytically active complex. Significantly, although dimeric RAG1 is the active form, it is highly susceptible to aggregation even at relatively low temperatures. The tendency for aggregation may be driven by small changes in protein conformation, although it is not associated with the global unfolding transition of RAG1. Significantly, the major V(D)J recombinase:RSS complex, formed after incubation at 37°C, contained RAG2 and a single dimer of RAG1, in contrast to lower temperatures where multiple protein-DNA complexes were formed. These results suggest that the RAG1 dimer is preferentially stabilized over higher order oligomers in the presence of RAG2. Overall, the characterizations of RAG1 in this study have resulted in a more complete picture of the biophysical properties of this protein, and will be valuable in further investigations of macromolecular interactions mediated by RAG1 during V(D)J recombination.

## Results and Discussion

### Temperature-dependent self-association properties of MCR1

Recombinant core RAG1, expressed and purified from bacteria as a fusion protein with maltose binding protein (MBP), was previously shown to be active in *in vitro *DNA cleavage assays when combined with RAG2 purified from 293T cells [24]. Recombinant core RAG1 (residues 384–1008) is produced in a soluble form at relatively high levels from this system, which has been useful for characterizing fundamental physicochemical properties of this protein. In contrast, production of the full length RAG1 protein in bacteria, to date, only yielded aggregated and inactive protein (unpublished data).

**Figure 1:**
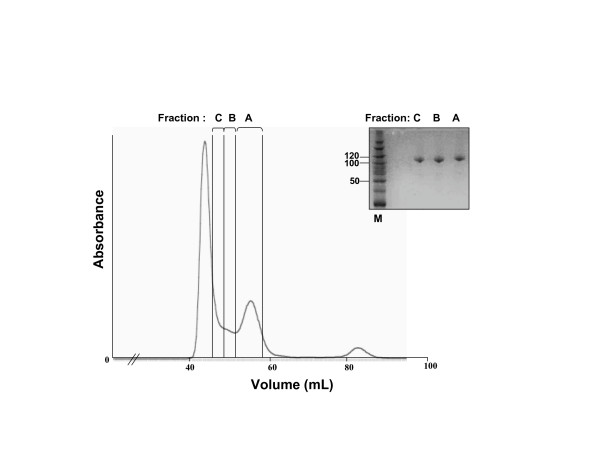
Preparative size exclusion chromatography of recombinant MCR1. A representative elusion profile of MCR1 from a 120 ml Superdex 200 gel filtration column is shown, which is the final chromatographic separation in MCR1 purification from bacterial cells. The elution positions of three separate sample pools used in this study, referred to as Fractions A, B, and C, are indicated with brackets. Collection of Fraction C commenced at the trailing edge of the void volume peak (at 1/3 peak height), and continued to a slight peak trough, where collection of Fraction B was begun. Fraction A was collected from the second peak trough through the trailing edge of the dimeric MCR1 peak (at 1/3 peak height). Inset: Coomassie Blue-stained SDS polyacrylamide gel (10%) of Fractions A, B, and C samples after purification and concentration to similar levels. The first lane contains molecular weight markers with the sizes of selected markers labeled (in kDa). The predicted molecular weight of monomeric MCR1 is 115.7 kDa.

The final purification step of the MBP-core RAG1 fusion protein (MCR1) was size exclusion chromatography (SEC) using a 120 ml Superdex 200 gel filtration column (see Methods). The SEC purification step was used to separate the dimeric form of MCR1 from aggregated material that eluted in the void volume. In addition to the dimeric form of the protein, higher order oligomeric forms of the protein were generated. In this study, and also as done previously [21], we focused on the characterization of three fractions of MCR1 (referred to as Fractions A, B, and C) that were pooled after the preparative SEC gel filtration step (Figure 1).

**Figure 2:**
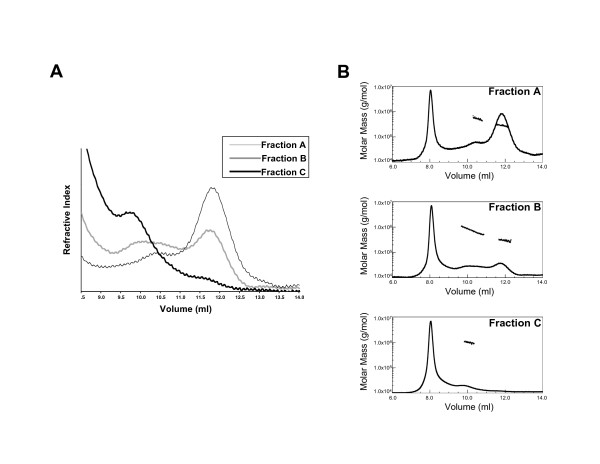
MALLS-SEC analyses of MCR1 Fractions A, B, and C. **(A) **Representative elution profiles of each fraction from SEC (20 ml Superdex 200 gel filtration column) and monitored by refractometry. Only the peaks retained during chromatographic separation are shown. Samples were incubated at 10°C for 30 min prior to analysis. **(B) **Molar mass distribution plots for each fraction. The elution profiles for each fraction in panel A are shown at full scale (including sample eluting in the void volume). The molar masses for sample eluting in the retained peaks are shown as filled circles. The molar mass is shown on the y-axis in log scale. Top panel: The elution profile of Fraction A consisted of two retained peaks with experimental masses of 520 kDa and 280 kDa, consistent with tetrameric and dimeric MCR1, respectively. (Due to incomplete resolution of peaks, the experimental masses tend to be ~10–20% larger than the predicted masses of the MCR1 oligomeric forms.) Middle panel: The retained peaks in elution profile of Fraction B included a broad peak, which was polydisperse in molecular mass, likely consisting of tetrameric and higher order oligomers of MCR1. A second retained peak was present, with experimental mass of 290 kDa, consistent with dimeric MCR1. Bottom panel: The elution profile of Fraction C consisted of a retained peak with experimental mass of 990 kDa, consistent with octameric MCR1.

The separate fractions were further characterized using multi-angle laser light scattering combined with SEC, referred to as MALLS-SEC. The SEC was performed using a 20 ml analytical Superdex 200 column that resulted in a significantly higher resolution for the separation of the MCR1 oligomeric species than the preparative SEC column, especially for Fraction C. The elution profiles of all three fractions obtained from MALLS-SEC at 10°C are shown in Figure 2. Consistent with our previous results [21], MCR1 self-associated as tetrameric and octameric forms, in addition to the dimeric form. Specifically, the elution profile of Fraction A showed two distinct peaks (resolved from the void volume) that corresponded in molecular mass to tetrameric [elution volume at maximum peak height (V_e _max) of 10.4 mL] and dimeric (V_e _max of 11.8 mL) MCR1 (Figure 2A &2B, top panel). The elution profile of Fraction B also showed two distinct peaks (Figure 2A); however, the first resolved peak eluted over a broad volume range (V_e _from 9.3 to 11.0 mL) and was more polydisperse in molecular mass (Figure 2B, middle panel), indicating co-elution of multiple oligomers (from tetramer to octamer). Lastly, a resolved peak (V_e _max of 9.8 mL) in the elution profile of Fraction C (Figure 2A) corresponded to octameric MCR1 as determined by MALLS-SEC analysis (Figure 2B, bottom panel). The elution profile of Fraction C also included a small peak at V_e _max of 11.7 mL. The concentration of protein eluting at this volume was too low to yield an accurate molecular mass; however, the V_e _max is consistent with dimeric MCR1. The first peak in each of the chromatograms in Figure 2B corresponded to protein that eluted in the void volume of the column, and likely included misfolded and/or non-specifically aggregated protein.

Previously, the relative proportions of the dimer, tetramer, and octamer forms of MCR1 as a function of ionic strength were examined [21]. At high ionic strengths (>0.5 M) it was found that the dimer form was favored; however, at near physiological ionic strengths (0.2 M) the total protein was comprised of an increased proportion of both the tetramer and octamer forms. While the different oligomeric MCR1 forms appeared to re-establish equilibria after changes in ionic strength, the redistribution occurred slowly (over ~48 hours). Therefore, the octamer could dissociate to smaller oligomers, albeit slowly, indicating that it was not formed by nonspecific aggregation of misfolded protein [21]. It is not likely MCR1 oligomers of a higher order than octamer exist, since protein that eluted in the void volume did not dissociate to lower oligomeric forms even at high ionic strengths [21].

**Figure 3:**
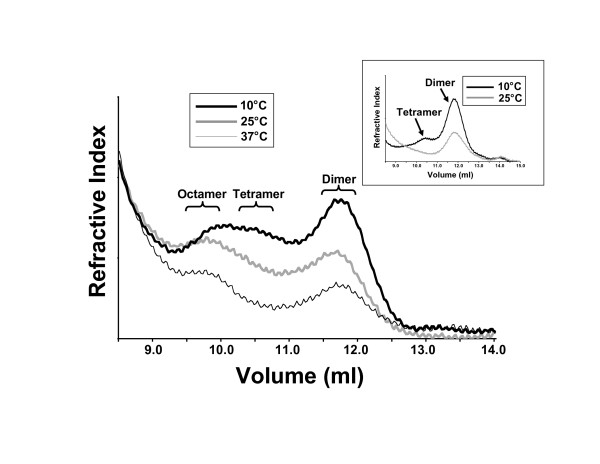
Temperature dependent oligomerization properties of MCR1. Overlay of multiple SEC profiles of Fraction B (performed as described in Figure 2A) with incubation for 30 min at the indicated temperatures prior to chromatography. Inset: Overlay of SEC profiles after incubation of Fraction A at 10 and 25°C.

Our findings of the three distinct oligomeric forms of RAG1 coupled with the contradictory reports of RAG1 stoichiometry in catalytically-active complexes [11-13] led us to further investigate the self-association properties of this protein. In this study we examined how the self-association properties of MCR1 varied with temperature (previous studies were performed at a single temperature with MCR1 samples incubated at 4°C prior to SEC [21]). Fractions A, B, and C were each incubated at the indicated temperatures for 30 min prior to analysis by SEC. With increasing temperature, the proportion of tetrameric protein appeared to decrease in Fraction B (Figure 3), as well as in Fraction A (Figure 3, inset). In Fraction A, the only retained peak at the higher temperatures contained dimeric MCR1. Likewise, in Fraction B, the proportion of tetrameric MCR1 in the broad peak at 10°C appeared to decrease at the higher temperatures, yielding resolved peaks with V_e _values consistent with the dimer and octamer forms only (Figure 3). It is likely that the reduction in the tetrameric form at temperatures higher than 10°C was due either to dissociation to the dimer form, or more likely, association to higher order oligomeric forms. With Fraction C, the octamer peak was present in the elution profiles over the entire temperature range of 10–37°C (not shown).

### The dimer form of RAG1 preferentially interacts with the RSS, RAG2, and catalyzes DNA cleavage

Of the resolved MCR1 species from SEC (excluding the void volume), Fractions A and C contained predominantly dimer or octamer species, respectively (Figure 2A). In contrast, Fraction B contained a mixture of oligomers, with the tetramer form only detectable at the lower temperatures (Figure 3). It is feasible that octameric RAG1 could contribute to RAG1 function in V(D)J recombination, due to its presence at physiological temperatures. To assess this possibility, we compared Fractions A and C in RAG1-related functions, including binding to 12-RSS, complex formation with RAG2, and DNA cleavage in a single 12-RSS cleavage assay.

**Figure 4:**
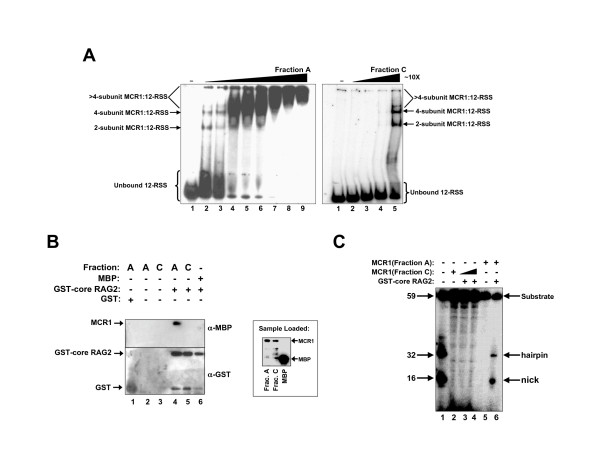
Dimeric MCR1, but not the octameric form, can maintain RAG1 related unctions. **(A) **Electrophoretic mobility shift assay demonstrating the interaction of MCR1 (Fraction A and Fraction C) with double-stranded WT 12-RSS. The radiolabeled substrate was titrated with increasing concentrations of either Fraction A (which was predominantly dimeric MCR1) or Fraction C (which was predominantly octameric MCR1). The protein concentrations ranged from 0.05 to 1 μM (lanes 2 to 9 for Fraction A) and 0.5 to 5 μM (lanes 2 to 5 for Fraction C). The samples were subjected to electrophoresis on a discontinuous (3.5/8%), non-denaturing polyacrylamide gel. Protein-DNA complexes consisting of either 2, 4, or >4 subunits of MCR1 bound to the 12-RSS are denoted by arrows. **(B) **Interaction between RAG2 and MCR1 (Fraction A and C). GST-core RAG2 (lanes 4–6) or GST (lane 1) was bound to glutathione-sepharose resin in the presence of MCR1 Fraction A (and in some cases Fraction C). The proteins eluted from the resin were resolved by SDS-PAGE and electrotransferred to polyvinylidene difluoride membranes. Left Panel: The interaction of GST and GST-core RAG2 with the glutathione resin was confirmed by the α-GST Western analysis blots. MCR1 fractions that interacted with GSTcore RAG2 are demonstrated by the α-MBP Western blot. Right Panel: α-MBP Western blot of Fractions A and C (lanes 1 and 2) and MBP (lane 3) at the loading concentrations used in the pull down assay. **(C) **DNA cleavage activity of MCR1 Fraction A and C. The proteins used are indicated above each lane. Radiolabeled double-stranded WT 12-RSS was incubated with MCR1 fractions and GST-core RAG2. DNA products were resolved on a 10% denaturing polyacrylmide gel. The protein concentrations were 10 μg/ml and 40 μg/ml (lanes 2, 3 and 4 for Fraction C) and 10 μg/ml (lane 5 and 6 for Fraction A). Concentration of GST-RAG2 used was 10 μg/ml. The reactions were in 5 mM Mn^2+^. The hairpin and nicked products are labeled. The percentages of nick and hairpin products relative to the total DNA in lane 6 were quantified at 16% and 6%, respectively.

To test for the affinity of binding to a single 12-RSS, electrophoretic mobility shift assays (EMSA) were performed. Increasing concentrations of MCR1 Fractions A and C were incubated with radiolabeled 12-RSS prior to electrophoresis under non-denaturing conditions. Fraction A (containing dimeric MCR1) formed multiple complexes with the RSS at protein concentrations of less than 0.2 μM (Figure 4A), consistent with our previous results in which we showed that MCR1 can form two major complexes with a single 12-RSS prior to saturation of the DNA [24]. In previous studies it was determined that the fastest migrating MCR1:RSS complex in the nondenaturing gel contained a single MCR1 dimer [24], whereas the slower migrating complex contained four MCR1 subunits bound to the 12-RSS [20].

The band labeled in Figure 4A as the 4-subunit MCR1:12-RSS complex may form from the interaction of the pre-formed tetramer or from two separate MCR1 dimers with the RSS. While there is some evidence that this complex may contain pre-formed tetramer [20], it is more likely that it is due to the binding of two separate dimers to the RSS, since (as shown in Figure 3) the pre-formed tetramer is less evident at 25°C (the typical incubation temperature used in the mobility shift experiments). However, in the event that two separate MCR1 dimers bind the RSS, it is not clear if both dimers form contacts with the RSS. Additionally, if the two dimers form contacts with each other on the RSS, then it is not possible to determine by EMSA if the interface is the same as that in the pre-formed tetramer. Due to these limitations, the slower mobility complex will be referred to as a 4-subunit MCR1:RSS complex rather than a tetrameric MCR1:RSS complex (Figure 4A).

A greater than 10-fold increase in the concentration of MCR1 Fraction C was required to shift an equivalent fraction of the RSS as compared to Fraction A (Figure 4A). It is likely, however, that the DNA binding observed in Fraction C was due to small amount of dimer present in this sample (see Figure 2A). Moreover, the relative migration of the protein-DNA complexes in the gel was the same between Fractions A and C. Since the fastest migrating band corresponds to dimeric MCR1 bound to the 12-RSS, this would be inconsistent with pre-formed MCR1 octamer forming a complex with the 12-RSS.

To compare the interaction of Fractions A and C with RAG2, a pull-down assay using core RAG2 fused to glutathione S-transferase (GST) was performed. GST-core RAG2 was incubated with the MCR1 fractions in the presence of glutathione-sepharose resin. After extensive washing of the resin with binding buffer, the proteins were released from the resin, resolved by SDS-PAGE, and analyzed by Western blot (Figure 4B). MCR1 Fraction A, but not Fraction C, bound to GST-core RAG2 under the conditions used here. It is possible that within the octameric form, the RAG1 subunits may be associated in a way that shields both the RSS and RAG2 binding sites.

As expected from the lack of RSS or RAG2 binding, MCR1 fraction C was inactive in a single RSS cleavage assay (when combined with RAG2). In this assay, both MCR1 fractions were incubated separately with GST-core RAG2 followed by the addition of double-stranded WT 12-RSS. As shown in Figure 4C (lane 6), dimeric MCR1 (Fraction A) was catalytically active and formed the hairpin and nicked products, whereas the octameric MCR1 (Fraction C) was inactive even with increased concentrations of protein from Fraction C (lanes 3&4).

It was somewhat surprising that Fraction C showed no evidence of DNA cleavage activity or interaction with RAG2, given that this fraction contained a small proportion of dimeric MCR1. It is possible that dissociation of the octameric protein to a dimeric form resulted in protein in an inactive conformation. Nevertheless, it is apparent that Fraction A isolated by preparative SEC contains the active form of MCR1.

### Conformational changes in RAG1 as a function of temperature

**Figure 5:**
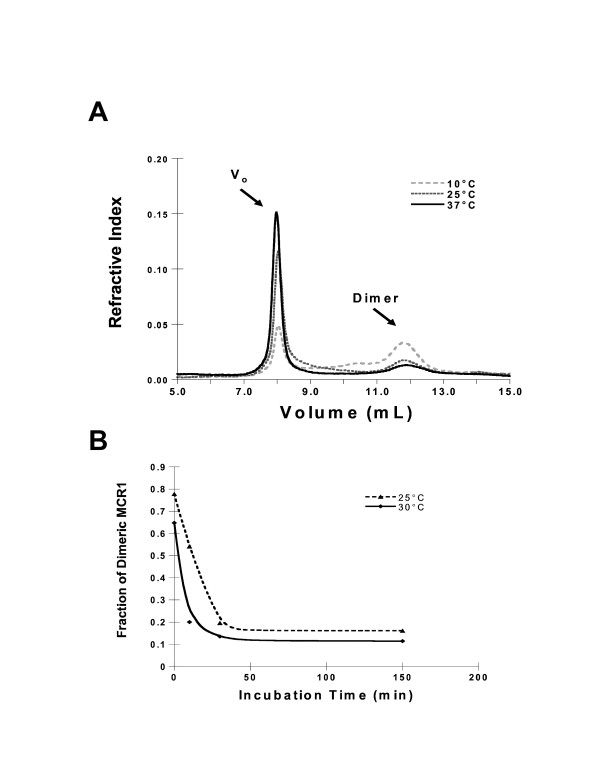
Analyses of temperature and temporal dependent aggregation of dimeric MCR1. **(A) **SEC elution profiles of Fraction A, including material eluting in the void volume (V_o_) after incubation for 30 min at 10, 25, and 37°C. **(B) **The fraction of MCR1 (Fraction A) remaining in the dimeric state after incubation at 0, 10, 30, or 150 min at either 25 or 30°C, as determined by peak integration of SEC elution profiles. Fraction A sample for the 0 min time point was at 4°C prior to SEC analysis.

Although the dimeric form of MCR1 was the most effective at carrying out RAG1 functions, the relative proportion of the dimeric form in Fraction A was substantially decreased with increasing temperature, with the majority of protein in an aggregated state at 25 and 37°C (Figure 5A). Specifically, ~60% of the total protein in Fraction A was dimeric at 10°C, whereas <10% was dimeric at temperatures of 25°C and greater, with the remainder aggregated in an inactive form. The aggregation occurred within an incubation period of 30 min at two temperatures tested, with no further aggregation occurring between 30 to 150 min incubation times (Figure 5B).

**Figure 6:**
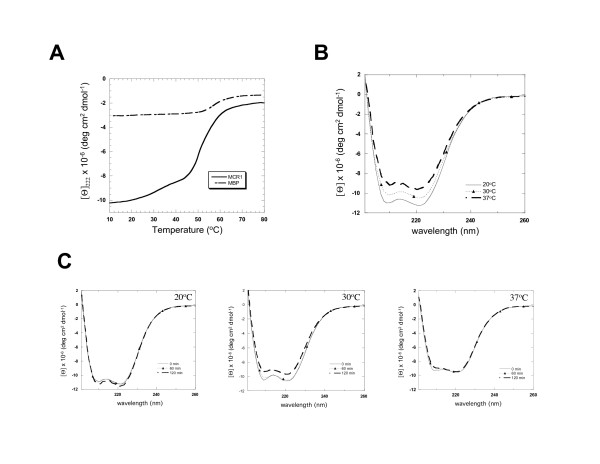
Circular dichroism spectroscopy of MCR1 as a function of temperature. **(A) **Thermal denaturation curves were recorded for MCR1 (—) and MBP (---). Denaturation curves are in units of molar ellipticity vs. temperature. **(B) **CD spectra of MCR1 samples after 60 min incubation periods at 20°C, 30°C, and 37°C. **(C) **CD spectra of MCR1 recorded with increasing incubation times (from 0 to 120 min) at 20°C, 30°C and 37°C. CD wavelength spectra in panels B and C are in units of molar ellipticity vs. wavelength.

To determine if the aggregation of dimeric MCR1 was due to protein unfolding, we measured the thermal denaturation of dimeric MCR1 (Fraction A) by circular dichroism (CD) spectroscopy. The protein showed two transitions with a minor transition occurring between 20 to ~37°C and a major transition at ~55°C (Figure 6A). Both transitions were irreversible under the conditions used here. Notably, the major protein unfolding transition occurred at higher temperatures (~50–60°C) than the range of interest (10–37°C). The denaturation curve of MBP alone was also measured, with the transition temperature for MBP unfolding at ~57°C, with a relatively constant CD signal from 10–50°C (Figure 6A). Therefore, it is consistent that the minor transition between 20–37°C is due to the RAG1 portion of MCR1, and thus this transition may reflect conformational changes that occur upon oligomerization of the protein.

A less likely possibility for the minor CD transition between 20–37°C could be due to a temperature dependent disruption in an interface formed between the core RAG1 and MBP portions of the fusion protein. A change in CD signal of the magnitude observed here would result from significant changes in the secondary structure of one or the other proteins upon complex dissociation. We believe such an interaction to be unlikely in this case since previous attempts to detect association between MBP and core RAG1 (after proteolytic cleavage from MBP) by various methods, such as chromatography and protein-protein crosslinking, were unsuccessful (not shown).

The CD spectra for MCR1 incubated at 20°C, 30°C, and 37°C for 60 min are shown in Figure 6B. At 10–20°C, MCR1 is predicted to contain 52% α-helices and 14% β-sheet, whereas at 37°C, the proportion of α-helical structure is decreased by 10%. Since the transition between 20–37°C was irreversible, there may be a time-dependent shift in the CD signal within this temperature range. Additionally, it was important to confirm that protein incubated at temperatures between 20–37°C did not enter into the major unfolding transition over time. To analyze time-dependent changes in the conformation within this temperature range, CD spectra were collected over a 2-hour incubation period at 20°C, 30°C, and 37°C. At temperatures representing the beginning and end of the first minor transition (20 and 37°C), there were no changes in either set of CD spectra over a 2 hr time period (Figure 6C, left and right panels). Therefore, MCR1 remained in its low temperature folded state at 20°C, while at 37°C the protein had obtained a stable intermediate conformation. After incubation at 30°C, the CD spectra changed slowly with time, with the final spectrum at 2 hr incubation matching the spectrum obtained at 37°C. This was expected, given the irreversibility of the minor transition. In conclusion, the majority of aggregation of dimeric MCR1 occurred between 20–37°C, which coincided with an irreversible minor transition in the thermal denaturation profile. The minor transition may be due to unfolding of a small region in core RAG1 that increases the propensity for nonspecific aggregation.

### Temperature dependent association of the V(D)J recombinase and the RSS

**Figure 7:**
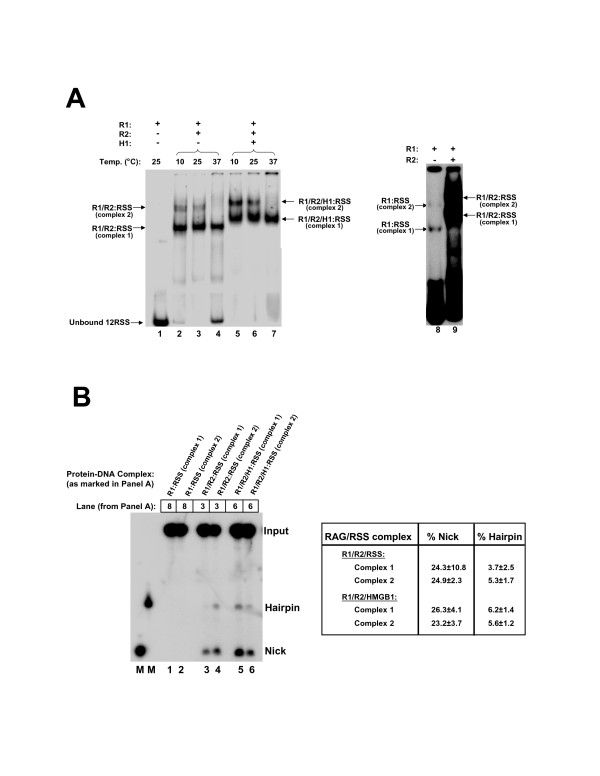
The V(D)J recombinase:12-RSS complex containing RAG2 and a single RAG1 dimer is preferentially formed at physiological temperature. **(A) **Left Panel: Radiolabeled 12-RSS was incubated with 0.04 μM MCR1 and 0.14 μM GST-core RAG2 (and 0.34 μM HMGB1 in some samples) at 10°, 25°, or 37°C, as indicated. The supershifted bands in lanes 2–4 that contain RAG2, and in lanes 5–7 that contain both RAG2 and HMGB1, are designated as complexes 1 and 2, respectively, with complex 1 containing 2 RAG1 subunits and complex 2 containing 4 RAG1 subunits. R1 and R2 refer to MCR1 and GST-core RAG2, respectively. H1 refers to HMGB1. Right Panel: Overexposure of autoradiogram to illustrate band positions of R1:RSS complexes (labeled as complex 1 and 2), relative to R1/R2:RSS complexes. Lanes 8 and 9 correspond to lanes 1 and 2 in the left panel. (It should be noted that in lane 8, R1:RSS complexes 1 and 2 correspond to the 2- and 4-subunit MCR1:12-RSS complexes denoted in Figure 4A.) **(B) **Left Panel: The supershifted RAG1:RAG2 complexes with the 12-RSS are catalytically active as determined by an in-gel cleavage assay (see Methods). The hairpin products in lanes 3 and 6 are more visible upon longer exposures of the gel (not shown). Complexes 1 and 2 refer to the bands as labeled in panel A. For example, lane 4 represents the cleavage products generated from the R1/R2 complex 2 in lane 3 of panel A. Right Panel: Quantification of the percentage of nick and hairpin products (from n = 3 in-gel cleavage assays) relative to the total amount of radioactivity in each lane.

In previous studies it was shown that RAG2 could bind to both the 2-subunit MCR1:12-RSS and the 4-subunit MCR1:12-RSS complexes following incubation at 25°C [20]. Since these complexes may be due to either one or two active RAG1 dimers bound to the 12-RSS, we asked if both of these complexes may be relevant to RAG1 function at 37°C. Here, we monitored the formation of the MCR1:RAG2:12-RSS complexes as a function of temperature. Upon incubation at 10 and 25°C, two complexes were formed (Figure 7A, lane 3), as has been previously observed under these conditions [20]. The addition of GST-core RAG2 substantially increased the level of protein-DNA complexes formed (lane 1 versus lanes 2–7), which may be due either to RAG2-induced conformational changes in RAG1 or to additional contacts formed between RAG2 and the RSS.

Significantly, upon incubation at 37°C, the higher mobility MCR1:GST-core RAG2 complex with 12-RSS (complex 1) was maintained, while the level of complex 2 was significantly diminished (Figure 7A, lane 4). There appears to be a slightly lower binding affinity overall at 37°C given the increased amount of unbound 12-RSS (lane 4). However, the addition of HMGB1 also showed decreased formation of the slower mobility complex 2 at 37°C, but with no significant increase in unbound 12-RSS (Figure 7A, lanes 5–7). Overall, in both lanes 4 and 7, there was robust formation of complex 1, but with little evidence of complex 2. This is in contrast to lower temperatures where both complexes are observed, particularly after saturation of the oligonucleotide duplex (as in Figure 7A). Complexes 1 and 2 were both catalytically active, with nicking and hairpin formation detected by an in-gel DNA cleavage assay (Figure 7B). In this assay, the gel slices containing Complexes 1 and 2 (formed after incubation at 25°C as in Figure 7A, lane 3) were tested for DNA cleavage activity. Moreover, it should be noted that since DNA cleavage activity must be performed at 37°C, it is possible that a RAG1 dimer could dissociate from Complex 2, effectively forming Complex 1.

**Figure 8:**
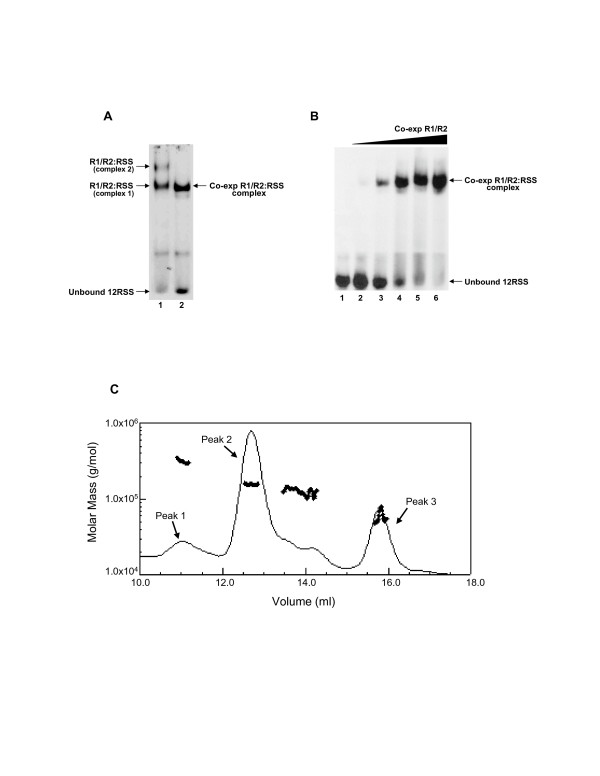
Co-expressed R1 and R2 form a single complex with the 12-RSS. **(A) **EMSA comparing complex formation of separately-expressed versus co-expressed (in 293T cells) of MCR1 and GST-core RAG2 with the 12-RSS. Lane 1 consists of the separately-expressed proteins and is analogous to lane 3 in Figure 7A. Lane 2 consists of co-expressed R1 and R2 proteins (at concentrations of 4 nM R1 and 2 nM R2). The protein-DNA complex in lane 2 coincides in migration position to complex 1 in lane 1. **(B) **Titration of increasing concentrations of co-expressed R1 and R2 proteins to the 12-RSS. The R1/R2 complex concentration (in terms of R1 concentration) varied from 1.7 nM in lane 2 to 28 nM in lane 6. **(C) **A representative molar mass distribution plot of GST-core RAG2 obtained from MALLS-SEC analysis. The molar masses for sample eluting in the retained peaks are shown as filled circles. The molar mass is shown on the y-axis in log scale. Experimental masses for protein eluting in Peaks 1 and 2 were at 314 kDa and 156 kDa, respectively, most consistent with tetrameric and dimeric GST-core RAG2 (predicted monomer molar mass at 68.9 kDa). The shoulder on Peak 2 consisted of protein that eluted with a molar mass of 127 kDa, and material eluting in Peak 3 was determined to be at a molar mass of 59 kDa.

MCR1 purified from mammalian cells showed similar results, as MCR1 and GST-core RAG2 protein co-expressed in 293T cells primarily formed complex 1 (with only low levels of complex 2 evident upon overexposure of the gel) with incubation at 25°C (Figure 8A). Further, complex 1 remained the predominant complex formed even after saturation of the 12-RSS substrate with increasing concentrations of the co-expressed RAG proteins (Figure 8B). Overall, it appears that complex 1 is the most stable RAG1:RAG2:RSS complex observed under various conditions, which is consistent with the findings from the physicochemical characterizations that the dimeric form of MCR1 is the relevant oligomer that functions in V(D)J recombination. We have previously assigned RAG1:RAG2:RSS complexes 1 and 2 (in Figure 7A) as GST-core RAG2 bound to the 2-subunit and 4-subunit MCR1:12-RSS complexes, respectively [20]. There is a possibility that complex 2 could be due to the binding of additional GST-core RAG2 subunits to the 2-subunit MCR1:12-RSS complex, which would correspond to the previously reported complexes "SC1" and "SC2" that contain 1 and 2 subunits of RAG2 bound to the RAG1 dimer:RSS complex, respectively [12]. However, we do not believe that this is the case, but rather, under the conditions used here, only one physiologically relevant RAG1:RAG2:12-RSS complex can form (complex 1 in Figure 7A). This may be due to the use of a GST tag on RAG2 in our system, which could preclude formation of an 'SC1' complex containing a single subunit of RAG2 given that the GST moiety is known to dimerize [25].

To test the oligomeric state of GST-core RAG2, MALLS-SEC was performed on the purified protein (Figure 8C). In summary, the major oligomeric form of GST-core RAG2 detected was dimer (eluting in Peak 2), with possibly minor amounts of tetramer (Peak 1), but no obvious monomeric species present. Specifically, protein eluting in the predominant peak (V_e _max of 12.7 mL; labeled Peak 2) was at a molar mass consistent with the dimer form. Material eluting in a shoulder of this peak (V_e _from 13.5 to 14.5 mL) was also at a molar mass consistent with dimeric GST-core RAG2. (Material eluting at this position may be due to an alternate conformation of the protein or to interactions with the column matrix.) Lastly, a peak with V_e _of 15.9 mL (Peak 3) yielded a molar mass of 59 kD. Peak 3 is likely due to the presence of a contaminating protein, which is evident on Coomassie blue-stained SDS polyacrylamide gels at ~55 kDa. The contaminating protein does not appear to be derived from either GST (at 26.3 kDa) or the core RAG2 portion (at 42.6 kDa) of the fusion protein. Moreover, protein eluting in Peak 3 could be not be detected by Western blotting against a monoclonal α-GST antibody (not shown). Finally, MCR1 combined with material eluting in Peak 2, but not Peak 3, resulted in the formation of complex 1 (as labeled in Figure 7A) by EMSA and formed hairpin products in DNA cleavage assays (not shown). As the protein concentration eluting in Peak 2 varied between 40–200 nM, which is within the range of protein concentration used in these studies (for example in Figure 7A), we surmise that GST-core RAG2 is in the dimeric state. These results are consistent with previous studies, which showed that dimeric GST unfolds in a two-state mechanism to denatured monomers, indicating that a monomeric folded form of GST is highly unstable [26].

## Conclusion

In this study, we have shown that self-association of RAG1 is temperature dependent from 10 to 37°C. Characterization of the temperature dependent variation in RAG1 oligomerization is essential, especially since the binding interactions of RAG1 with RSS have typically been examined at either 4°C or 25°C, but DNA cleavage activity is monitored at 37°C. In summary, we found that the relative proportions of each RAG1 oligomer changed significantly as the temperature varied from 10–37°C. While the dimer and octamer forms were observed over this entire temperature range, the tetrameric protein was only detected at the lowest temperatures studied. Although both the RAG1 dimer and octamer forms were present at physiological temperature, only the dimer-containing sample was active in DNA binding, RAG2 interaction, and in DNA cleavage. In contrast, the octamer-containing sample appeared to show no significant activity in any of the assays tested, indicating that this higher order pre-formed oligomer cannot contribute to RAG1 activity in V(D)J recombination.

We conclude that the single RAG1 dimer:RSS complex is the physiologically relevant moiety, since this complex with RAG2 incorporated is preferentially formed at 37°C with separately expressed proteins, and is the predominant complex formed with co-expressed proteins. Altogether, these results show that at physiological temperature only a single dimer of RAG1 is stably incorporated into the catalytically-active V(D)J recombinase:RSS complex. Thus, we are in agreement with previous reports that the stoichiometry of the physiologically-relevant V(D)J recombinase consists of two RAG1 subunits [11,12].

Significantly, our finding that dimeric RAG1 aggregates upon a minor conformational change in the temperature range of 25 to 37°C was previously not known. Understanding such physicochemical properties of RAG1 under different conditions is necessary for the interpretation of experiments conducted in solution, and may be useful in determining conditions to perform structural studies of this protein.

## Methods

### Protein expression and purification

The MBP-core RAG1 (MCR1) fusion protein was expressed in *Escherichia coli *and purified as described previously [18]. MBP was expressed and purified as described [20]. GST-core RAG2, either alone or co-expressed with MCR1, was transiently expressed and purified from 293T cells as previously reported [16,27].

The concentrations of MCR1 and MBP purified from *E. coli *were determined from the absorbance at 280 nm using extinction coefficients of 129.5 and 66.5 mM^-1^cm^-1^, respectively. The concentrations of proteins purified from 293T cells were determined by quantitative Western blotting as previously described [27].

### Multi-angle laser light scattering with size-exclusion chromatography

Multi-Angle Laser Light scattering with Size-Exclusion Chromatography (MALLS-SEC) was accomplished using a DAWN DSP laserphotometer coupled with an Optilab DSP interferometric refractometer (Wyatt Technology; Santa Barbara, CA) and combined in-line with a Superdex 200 gel filtration column (GE Healthcare; Piscataway, NJ).

Before each injection MCR1 was incubated at 10, 25, or 37°C as indicated, followed by chromatographic separation at room temperature. The column buffer for MCR1 MALLS-SEC experiments was comprised of 20 mM Tris, pH 8.0; 50 μM ZnCl_2_; 5 mM β-mercaptoethanol; and 200 mM NaCl. For each experiment, between 0.06 and 0.13 mg of MCR1 was injected. Prior to SEC, GST-core RAG2 samples (at 0.01–0.03 mg) were kept at 4°C. The column buffer for GST-core RAG2 MALLS-SEC analysis was 25 mM Tris, pH 8.0; 150 mM KCl; and 2 mM dithiothreitol. Analysis of MALLS-SEC data was done as previously described [28].

### Circular dichroism spectroscopy

The circular dichroism (CD) spectroscopy experiments were performed using a JASCO J715 spectropolarimeter with a PTC-348WI peltier temperature controller (Jasco, Corp.; Tokyo, Japan). The parameters for the spectra measurements are: 1.0 nm bandwidth, 1 nm resolution, 16 sec. response time, and 5 accumulations. The protein concentration in each sample was in the range of 1.7–2.0 μM and the buffer consisted of 20 mM Tris pH 8.0, 200 mM NaCl. Analysis of secondary structural content was done as previously described [20,21].

Thermal denaturation experiments were performed in 10 mM Tris pH 8.0, 5 mM MgCl_2_, and 100 mM NaCl using the same protein concentrations as the wavelength scan described above. The CD signal was detected at 222 nm, and the parameters were as follows: 10–80°C temperature range, 1.0 nm bandwidth, 0.2°C resolution, and a response time of 16 sec.

### Electrophoretic mobility shift assay (EMSA)

An oligonucleotide duplex containing a 12-RSS, commercially synthesized and PAGE-purified (Integrated DNA Technologies), was used as the DNA substrate in the EMSA experiments. The sequence of the 12-RSS containing oligonucleotide duplex has been previously reported [29]. The 12-RSS substrate was labeled with ^32^P at the 5'-end of the top strand using γ^32^P-ATP and T4 polynucleotide kinase, and the double-stranded substrate was prepared by annealing the top strand with its respective complementary oligonucleotide.

EMSA was performed as described previously [20]. The binding buffer contained 20 mM Tris, pH 8.0; 5 mM MgCl_2_; 6% glycerol; 100 mM NaCl; and 2 mM dithiothreitol. In samples containing both RAG1 and RAG2, the binding buffer contained 2 mM CaCl_2 _in place of MgCl_2 _to prevent DNA cleavage. Prior to loading the samples into the gel, samples were incubated for 30 minutes at room temperature unless otherwise specified.

### Pull-down assay

Prior to the addition of the RAG proteins, glutathione-sepharose 4B resin (GE Healthcare; Piscataway, NJ) was blocked with 1 mg/ml bovine serum albumin in interaction buffer (20 mM Tris-HCl, pH 8.0, 0.2 M NaCl, 10% glycerol, 10μM ZnCl_2_, and 5 mM β-mercaptoethanol) for 45 min at 4°C, followed by washing the resin three times with interaction buffer. Subsequently, MCR1 Fractions A or C (at 0.1 μM) or MBP (at 0.5 μM) was combined with GST-core RAG2 or GST (at 0.15 μM), and the proteins incubated with resin in the interaction buffer for 1 h at 4°C. Following three washes with the interaction buffer, the bound proteins were eluted from the resin with SDS loading buffer, resolved by SDS-PAGE (10% polyacrylamide gel), and electrotransferred to polyvinylidene difluoride membrane. Western analysis was done for both MBP and GST proteins as previously described [18].

### Single RSS DNA cleavage assay

The single RSS cleavage assay was performed in a total volume of 10 μl with a buffer composed of 10 mM Tris (pH 8.0), 2 mM dithiothreitol, 6% glycerol, 100 mM NaCl and 5 mM MnCl_2_. MCR1 samples were incubated with GST-core RAG2 for 30 min at 4°C, followed by addition of 1 nM ^32^P-labeled double-stranded wild type (WT) 12-RSS and incubation for 2 hrs at 37°C. The remaining procedure was as described previously [30].

### In-gel DNA cleavage assay

The in-gel cleavage assay was done as previously described [31]. In summary, samples were electrophoretically separated by EMSA as described above. After electrophoresis, the nondenaturing polyacrylamide gel was submerged in reaction buffer (10 mM Tris, pH8, 100 mM NaCl, and 5 mM MnCl_2_) at 37°C for 1.5 hour. Next the gel was electro-transferred to DEAE membrane at 4°C overnight (50 V, 100 mA). To visualize the supershifted bands, the membrane was exposed to autoradiographic film overnight at 4°C, and washed in sterile wash buffer (50 mM NaCl, 10 mM Tris pH 7.5, 1 mM EDTA). Using the autoradiographic film as a guide, the supershifted bands were cut from the membrane and incubated with DNA elution buffer (1 M NaCl, 10 mM Tris pH 7.5, 1 mM EDTA) at 65°C for 30 min. The eluted DNA was filtered from the DEAE membrane using 0.22 μm Cellulose Acetate Spin X Centrifuge Tubes. After elution, the DNA was precipitated with ethanol and resuspended in 10 μL TE/Formamide loading dye (50% TE + 50% formamide loading dye). Samples were heated 2 min at 72°C, followed by separation on a 10% denaturing polyacrylamide gel. Bands corresponding to nick and hairpin cleavage products were visualized by autoradiographic analysis.

## Authors' contributions

PD and KKR designed the study and KKR was responsible for study coordination. The MALLS-SEC results in Figures 1, 2, 3, and the protein-protein interaction assay and the protein- DNA interaction and cleavage assays in Figure 4 were performed and analyzed by PD. The MALLS-SEC results in Figure 5, and the gel mobility shift assays in Figure 8, were performed and analyzed by SZ. The circular dichroism experiments were performed and analyzed by LMG, with assistance from SZ. LJG made the initial observations on the temperature dependent self-association properties of MCR1 and performed the EMSA experiment shown in Figure 7A. The in-gel DNA cleavage assay was done by MMP. PD and KKR drafted the manuscript, and all authors critically read and approved the final manuscript. The MALLS-SEC experiment in Figure 8C was performed by LJG and analyzed by LMG. All authors have read and approved the final manuscript.

## References

[B1] Fugmann SD, Lee AI, Shockett PE, Villey IJ, Schatz DG (2000). The RAG proteins and V(D)J recombination: complexes, ends, and transposition. Annu Rev Immunol.

[B2] Gellert M (2002). V(D)J recombination: RAG proteins, repair factors, and regulation. Annu Rev Biochem.

[B3] McBlane JF, van Gent DC, Ramsden DA, Romeo C, Cuomo CA, Gellert M, Oettinger MA (1995). Cleavage at a V(D)J recombination signal requires only RAG1 and RAG2 proteins and occurs in two steps. Cell.

[B4] Lieber MR, Ma Y, Pannicke U, Schwarz K (2003). Mechanism and regulation of human nonhomologous DNA end-joining. Nat Rev Mol Cell Biol.

[B5] Jones JM, Gellert M (2002). Ordered assembly of the V(D)J synaptic complex ensures accurate recombination. EMBO J.

[B6] Eastman QM, Schatz DG (1997). Nicking is asynchronous and stimulated by synapsis in 12/23 rule-regulated V(D)J cleavage. Nucleic Acids Res.

[B7] Yu K, Lieber MR (2000). The nicking step in V(D)J recombination is independent of synapsis: implications for the immune repertoire. Mol Cell Biol.

[B8] Curry JD, Geier JK, Schlissel MS (2005). Single-strand recombination signal sequence nicks in vivo: evidence for a capture model of synapsis. Nat Immunol.

[B9] West RB, Lieber MR (1998). The RAG-HMG1 complex enforces the 12/23 rule of V(D)J recombination specifically at the double-hairpin formation step. Mol Cell Biol.

[B10] Swanson PC (2002). Fine Structure and Activity of Discrete RAG-HMG Complexes on V(D)J Recombination. Mol Cell Biol.

[B11] Bailin T, Mo X, Sadofsky MJ (1999). A RAG1 and RAG2 tetramer complex is active in cleavage in V(D)J recombination. Mol Cell Biol.

[B12] Swanson PC (2002). A RAG-1/RAG-2 tetramer supports 12/23-regulated synapsis, cleavage and transposition of V(D)J recombination signals. Mol Cell Biol.

[B13] Mundy CL, Patenge N, Matthews AGW, Oettinger MA (2002). Assembly of the RAG1/RAG2 synaptic complex. Mol Cell Biol.

[B14] Landree MA, Wibbenmeyer JA, Roth DB (1999). Mutational analysis of RAG1 and RAG2 identifies three catalytic amino acids in RAG1 critical for both cleavage steps of V(D)J recombination. Genes Dev.

[B15] Kim DR, Dai Y, Mundy CL, Yang W, Oettinger MA (1999). Mutations of acidic residues in RAG1 define the active site of the V(D)J recombinase. Genes Dev.

[B16] Spanopoulou E, Zaitseva F, Wang F-H, Santagata S, Baltimore D, Panayotou G (1996). The homeodomain region of Rag-1 reveals the parallel mechanisms of bacterial and V(D)J recombination. Cell.

[B17] Difilippantonio MJ, McMahan CJ, Eastman QM, Spanopoulou E, Schatz DG (1996). RAG1 mediates signal sequence recognition and recruitment of RAG2 in V(D)J recombination. Cell.

[B18] Arbuckle JL, Fauss LJ, Simpson R, Ptaszek LM, Rodgers KK (2001). Identification of two topologically independent domains in RAG1 and their role in macromolecular interactions relevant to V(D)J recombination. J Biol Chem.

[B19] Lin WC, Desiderio S (1994). Cell cycle regulation of V(D)J recombination-activating protein RAG-2. Proc Natl Acad Sci USA.

[B20] Godderz LJ, Rahman NS, Risinger GM, Arbuckle JL, Rodgers KK (2003). Self-association and conformational properties of RAG1: Implications for formation of the V(D)J recombinase. Nucleic Acids Res.

[B21] Godderz LJ, Rodgers KK (2004). RAG1 oligomerization states and secondary structural properties: An initial characterization of V(D)J recombinase complex formation. Spectrosc – Int J.

[B22] Hickman AB, Perez ZN, Zhou L, Musingarimi P, Ghirlando R, Hinshaw JE, Craig NL, Dyda F (2005). Molecular architecture of a eukaryotic DNA transposase. Nat Struct MolBiol.

[B23] Zhou L, Mitra R, Atkinson PW, Hickman AB, Dyda F, Craig NL (2004). Transposition of hAT elements links transposable elements and V(D)J recombination. Nature.

[B24] Rodgers KK, Villey IJ, Ptaszek L, Corbett E, Schatz DG, Coleman JE (1999). A dimer of the lymphoid protein RAG1 recognizes the recombination signal sequence and the complex stably incorporates the high mobility group protein HMG2. Nucleic Acids Res.

[B25] Lim K, Ho JX, Keeling K, Gilliland GL, Ji X, Ruker F, Carter DC (1994). Three-dimensional structure of Schistosoma japonicum glutathione S-transferase fused with a sixamino acid conserved neutralizing epitope of gp41 from HIV. Protein Sci.

[B26] Kaplan W, Husler P, Klump H, Erhardt J, Sluis-Cremer N, Dirr H (1997). Conformational stability of pGEX-expressed Schistosoma japonicum glutathione S-transferase: a detoxification enzyme and fusion-protein affinity tag. Protein Sci.

[B27] Rahman NS, Godderz LJ, Stray SJ, Capra JD, Rodgers KK (2006). DNA cleavage of a cryptic recombination signal sequence by RAG1 and RAG2: Implications for partial VH gene replacement. J Biol Chem.

[B28] Godderz LJ, Peak MM, Rodgers KK (2005). Analysis of biological macromolecular assemblies using static light scattering methods. Curr Org Chem.

[B29] Peak MM, Arbuckle JL, Rodgers KK (2003). The central domain of core RAG1 preferentially recognizes single-stranded recombination signal sequence heptamer. J Biol Chem.

[B30] De P, Peak MM, Rodgers KK (2004). DNA cleavage activity of the V(D)J recombination protein RAG1 is autoregulated. Mol Cell Biol.

[B31] Bergeron S, Anderson DK, Swanson PC (2006). RAG and HMGB1 proteins: purification and biochemical analysis of recombination signal complexes. Methods Enzymol.

